# Exposure to sequestered self-antigens *in vivo* is not sufficient for the induction of autoimmune diabetes

**DOI:** 10.1371/journal.pone.0173176

**Published:** 2017-03-03

**Authors:** Nobuyuki Ono, Kiichi Murakami, Olivia Chan, Håkan Hall, Alisha R. Elford, Patty Yen, Thomas Calzascia, David M. Spencer, Pamela S. Ohashi, Salim Dhanji

**Affiliations:** 1 Department of Rheumatology, Faculty of Medicine, Saga University, Saga, Japan; 2 Campbell Family Institute for Breast Cancer Research, Princess Margaret Cancer Centre, Departments of Medical Biophysics and Immunology, Toronto, Ontario, Canada; 3 Department of Pathology and Immunology, Baylor College of Medicine, Texas Medical Center, Houston, Texas, United States of America; 4 Bellicum Pharmaceuticals, Inc. Houston, Texas, United States of America; Universite Paris-Sud, FRANCE

## Abstract

Although the role of T cells in autoimmunity has been explored for many years, the mechanisms leading to the initial priming of an autoimmune T cell response remain enigmatic. The ‘hit and run’ model suggests that self-antigens released upon cell death can provide the initial signal for a self-sustaining autoimmune response. Using a novel transgenic mouse model where we could induce the release of self-antigens via caspase-dependent apoptosis. We tracked the fate of CD8+ T cells specific for the self-antigen. Our studies demonstrated that antigens released from apoptotic cells were cross-presented by CD11c+ cells in the draining lymph node. This cross-presentation led to proliferation of self-antigen specific T cells, followed by a transient ability to produce IFN-γ, but did not lead to the development of autoimmune diabetes. Using this model we examined the consequences on T cell immunity when apoptosis was combined with dendritic cell maturation signals, an autoimmune susceptible genetic background, and the deletion of Tregs. The results of our study demonstrate that autoimmune diabetes cannot be initiated by the presentation of antigens released from apoptotic cells *in vivo* even in the presence of factors known to promote autoimmunity.

## Introduction

Over the years, the field of autoimmunity has gained insights into mechanisms of tolerance, regulatory pathways and genes that have an impact on the development of autoimmunity. However, the underlying events that lead to the initiation of an autoimmune T cell response remain unclear. One mechanism that has been proposed is known as the ‘hit and run hypothesis’[1, 2], which suggests that infection, trauma, or injury to a particular tissue leads to cell death and the release of normally sequestered self-antigens. This process is believed to be a key event that initiates an autoimmune response that amplifies over time through epitope spreading and other mechanisms to result in autoimmunity.

One of the events, or ‘hits’, that leads to the initial release of self-antigens may be programmed cell death within a tissue or organ. There are different forms of programmed cell death including necroptosis, pyroptosis and apoptosis. Necroptosis is lytic cell death and a regulated form of necrosis that is induced by death receptors such as TNF receptor. After receptor-interacting protein kinase 1 (RIPK1) and RIPK3 activation, mixed lineage kinase domain-like protein (MLKL) is phosphorylated and this leads to necroptosis. Pyroptosis is mediated through the activation of caspase-1 and caspase-11 and is usually associated with the release of inflammatory cytokines, IL-1β and IL-18. Both necroptosis and pyroptosis cause ruptures in the cell membrane and results in the release of intracellular components (including damage-associated molecular patterns (DAMPs)) into the extracellular space which can trigger an inflammatory response [3, 4].

Apoptosis, on the other hand, is a non-lytic form of cell death and has been known to contribute to tissue turnover and the maintenance of homeostasis. The extrinsic and intrinsic signaling pathways of apoptotic cells trigger the activation of effector caspases such as caspase-3, -6 and -7 and induce morphological and functional changes. Apoptotic cells are cleared within minutes—engulfed by phagocytes such as macrophages or dendritic cells (DCs)–thereby preventing the release of DAMPs such as heat shock proteins (HSPs), the chromatin protein HMGB1 or uric acid [5, 6]. Studies have demonstrated that DC maturation does not occur upon encountering antigens released by apoptosis, and as a consequence, T cells specific for these antigens are tolerized by various mechanisms [7–11]. Furthermore, the uptake of apoptotic cells has been shown to actively suppress the expression of pro-inflammatory mediators or induce the expression of anti-inflammatory proteins in phagocytes [10, 12–15].

However, several reports have demonstrated that apoptotic cell death can create a pool of normally sequestered self-antigens which can be presented to T cells by antigen presenting cells (APCs) in the lymph node draining the organ. The normal physiological process of neonatal islet apoptosis suggest that this is a key event that leads to the presentation of islet antigens and the induction of autoimmunity in animal models of diabetes [16–18]. Apoptotic cells which arise from certain types of anti-cancer treatments have also been documented to induce an immune response [19–21]. Thus, under certain conditions, apoptosis has the potential to activate immune cells and a number of parameters which contribute to the immunogenicity of apoptotic cells [22, 23].

In the current study we set out to examine whether the sterile release of antigens by apoptosis could initiate autoimmune diabetes in the presence of various factors which could contribute autoimmunity. We have developed a novel model whereby we can specifically induce apoptosis in the β-islet cells of the pancreas *in vivo* without the use of cytotoxic drugs and associated inflammation. The induction of apoptosis in this model leads to the cross-presentation of β-islet antigens in the pancreatic draining lymph node to T cells by CD11c+ cells. The C57Bl/6 mouse strain expressing LCMV glycoprotein (gp) in β-islets have been widely studied as a virus-induced diabetes model and the non-obese diabetic (NOD) mice are known as a spontaneous type 1 diabetes model. Thus, the consequences of β-cell apoptosis and the induction of diabetes were evaluated in both strains. Our results suggest that antigens derived from apoptotic cells are capable of activating autoreactive CD8 T cells but is insufficient to promote autoimmune diabetes. Even in the presence of APC maturation signals or inflammatory conditions, a brief exposure of CD8 T cells to antigen from apoptotic cells is not sufficient to trigger a self-sustained autoimmune response in the pancreas. The results of this study provide evidence that the release of self-antigen from apoptotic cells can be presented to T cells, but this encounter ultimately leads to T cell tolerance and is not sufficient to induce self-sustaining autoimmune diabetes.

## Materials and methods

### Mice and blood glucose monitoring

P14 transgenic mice and virus-induced diabetes model, RIP-gp mice, were generated as previously described [24, 25]. C57BL/6 and spontaneous type 1 diabetes model, NOD mice, were purchased from Taconic. DEREG mice (C57Bl/6 background) were a kind gift from Tim Sparwasser. CD45.1, CD90.1 and CD11c-DTR mice were obtained from The Jackson Laboratory. To generate the RIP-iCasp-3 mice, iCaspase-3 cDNA was inserted into Xba1 site of the pINS vector to drive expression under the control of rat insulin 2 promoter. A fragment of RIP-iCasp-3 cassette was microinjected into the pro-nuclei of C57Bl/6 fertilized eggs. Four independent lines were produced. Blood glucose concentrations were determined (2–3 times per week) using Accu-chek III Glucometers and Chemstrips (Roche) and considered mice diabetic after two consecutive measurements of >15 mM. All mice were maintained at the Ontario Cancer Institute (Toronto, Canada) in accordance with approved protocols (AUP929) and were euthanized by carbon dioxide.

### Immunohistochemistry

Pancreata were frozen in OCT compound or fixed with 2% paraformaldehyde. Sections were stained with goat anti-human caspase-3 antibody (sc-1226; Santa Cruz Biotechnology) or rat anti-mouse CD8 antibody (YTS169).

### Adoptive transfer experiments

Single cell suspensions were prepared from spleens and lymph nodes of CD90.1+ P14 TCR transgenic mice or P14 TCR transgenic mice with CD45.1+ subtype, and CD8+ T cells were purified using CD8+ T cell selection kit (Miltenyi Biotech) and a magnetic activated cell sorter (Miltenyi Biotech). For adoptive transfer, purified cells were labeled with CFSE (5-[and-6]-carboxyfluorescein diacetate succinimidyl ester) (Sigma). 2.5-5x10^6^ purified labeled P14 cells were transferred intravenously into mice which were treated 24 hours later with AP20187 (Clontech or ARIAD Pharmaceutical) injected intraperitoneally. 5 days after injection, organs were harvested and stained for flow cytometric analysis.

### Tolerance experiment

3x10^6^ purified CFSE-labeled P14 cells were transferred to RIP-iCasp3.4/gp mice and were injected with AP20187 the following day. 5 days after drug injection draining lymph nodes of 6 mice were pooled and the P14 cells were sorted based on their CFSE profile. LCMV-activated CD8+ T cells were purified from P14 mice 3 days after viral infection using CD8+ T cell selection kit (Miltenyi Biotech). The purity of the sorted cells was greater than 95%. 5000 sorted cells were transferred to naïve C57Bl/6 mice which were infected with 2000 pfu of LCMV Armstrong strain (i.v.) the following day. 8 days after infection, splenocytes from the infected mice were stained with gp33-41(KAVYNFATM), gp276-286 (SGVENPGGYCL), np396-404 (FQPQNGQFI) tetramers (NIH Tetramer Core Facility) and used as effectors in an *ex vivo* CTL assay.

### Cytotoxic T lymphocyte assay

EL-4 target cells were pulsed with either MB6, np396, or gp33 peptides for 90 minutes in the presence of 400 μCi/ml ^51^Cr (Perkin Elmer) in IMDM supplemented with 10% FCS. Cells were washed and 10^4^ cells/well were transferred to U-bottom 96 well plate. Splenocytes from LCMV-infected mice were serially diluted and mixed with peptide pulsed target cells. Plates were incubated for 5 hours at 37°C. 70 μl of supernatant was counted from each well using a Wallac Wizard counter (Perkin Elmer). Maximal release was induced adding 1 M HCl to target cells. Percent specific lysis was calculated as (c.p.m. sample release—c.p.m. spontaneous release) / (c.p.m. maximum release—c.p.m. spontaneous release) x 100%.

### Intracellular cytokine staining

To examine IFN-γ production in transferred P14 T cells, cells from the lymph nodes were stimulated with 10^−7^ M gp33 peptide and 1μl/ml of Golgi-Plug solution (BD Biosciences) for 6 hours. Recovered cells were stained with anti-CD45.1-PE mAb (eBioscience), anti-CD8α-PerCP mAb (eBioscience) and anti-Va2-FITC mAb (BD Biosciences). Surface stained cells were permeabilized and stained with anti IFN-γ-APC mAb (eBioscience) by using the Cytofix/Cytoperm Plus kit (BD Biosciences). Diphtheria toxin was purchased from Sigma. Cells were analyzed by FACS Caliber or FACS Canto (BD Biosciences). Flow cytometry data were analyzed using FlowJo software (Tree Star).

### Pancreatic digestion and lymphocyte enumeration

After each pancreas was digested by 0.7 mg/ml of collagenase D (Roche) and 10 U/ml of DNase-1 (Roche), tissue debris was removed by density gradient centrifuge. Infiltrated cells were stained with anti-CD45.1-PE mAb, CD62L-PECy7 mAb, CD8α-APC mAb, CD4-APCCy7 (eBioscience) and 7-AAD (BD Biosciences).

### Statistics

Statistical significance between groups was determined by a two-tailed unpaired Student *t* test using GraphPad Prism (GraphPad Software).

## Results

### Drug induced apoptosis leads to release of detectable antigen from islet cells

**Fig 1:**
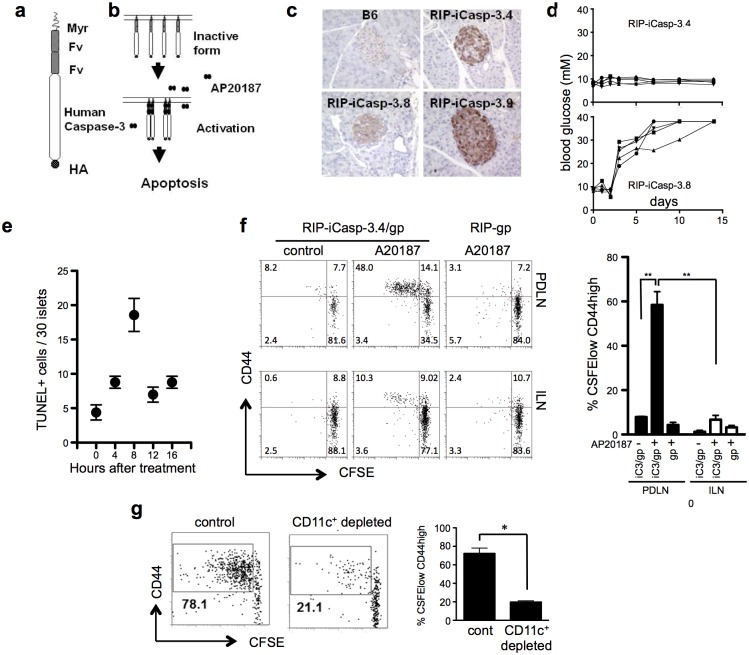
Induction of apoptosis in the RIP-iCasp-3 transgenic model leads to antigen specific T cell proliferation. (a) The inducible caspase-3 construct (iCasp-3) includes a human pro-caspase-3 DNA fused with a myristoylation sequence (Myr), two modified FK506 binding domains (Fv) and a hemagglutinin antigen (HA). (b) Schematic representation of how apoptosis is induced. The addition of the dimerizing agent AP20187 induces dimerization of the iCasp-3 construct and activation of caspase-3 with the subsequent induction of apoptosis. The rat insulin promoter (RIP) was used to express the iCasp-3 construct and transgenic mice were generated that express iCasp-3 in the β-islet cells of the pancreas. (c) Expression was examined by histology using an antibody specific for human caspase-3. (d) 2 mg/kg of A20187 was given by i.p. to RIP-iCasp3.4 and RIP-iCasp3.8 mice (3 times, 24 hours apart) and blood glucose levels were measured. (e) The pancreas sections from RIP-iCasp3.4 mice treated with 2 mg/kg AP20187 were analyzed by TUNEL staining at the indicated time points. (f) Purified CD45.1+ P14 CD8 were labeled with CFSE and transferred to either RIP-iCasp-3.4/gp or RIP-gp mice which were injected with 2 mg/kg AP20187 the following day. 5 days after drug injection, proliferation of P14 cells was analyzed in the pancreatic draining (PDLN) or inguinal (ILN) lymph node. (g) RIP-iCasp-3.4/gp/CD11c-DTR mice were treated with diphtheria toxin to deplete CD11c+ cells prior to AP20187 injection. Dot plots depict the proliferation of CFSE-labeled P14 cells 5 days post injection with AP20187. Data are representative of at least 5 mice per condition from independent experiments. Error bars in bar graph show S.E.M. (**p < 0.01, *p < 0.05).

In order to examine whether apoptosis can lead to self-antigen presentation and initiate autoimmune diabetes we generated a model where we could control the induction of β-islet cell apoptosis and subsequent release of β-islet cell antigens. We utilized a chemically induced dimerization technology, where a dimeric FK506 analogue, AP20187, is able to crosslink two proteins that are modified to contain a high affinity AP20187-binding domain, FKBP12v36 (“F_v_”). Two F_v_ domains were linked downstream of a myristoylation sequence, and fused with human caspase-3 and a hemagglutinin antigen (HA) epitope (Fig 1a). The drug AP20187 induces dimerization of the Fv/caspase-3 fusion protein and leads to the induction of apoptosis via caspase-3 dependent pathways (Fig 1b). This model has been shown previously to be effective *in vivo* [26]. The inducible caspase-3 construct (iCasp-3) was expressed as a transgene under the control of the rat insulin promoter (RIP); thereby limiting the expression of the construct to the β-islet cells of the pancreas. Antigen release in these RIP-iCasp-3 mice can therefore be tightly regulated, and the onset of autoimmune diabetes in these animals could be assessed by elevated levels of blood glucose. Three different founder lines, iCasp-3.4, iCasp-3.8, and iCasp-3.9, were generated on a C57Bl/6 background and analyzed for expression of the transgene. Pancreatic sections stained with a monoclonal antibody specific for human caspase-3 showed that expression of the transgene was restricted to the β-islets of the pancreas (Fig 1c).

To evaluate whether the administration of AP20187 could induce apoptosis *in vivo*, several experiments were conducted. First, RIP-iCasp-3 transgenic mice were given 2 mg/kg AP20187, 3 times intraperitoneally, 24 hours apart, and blood glucose levels were followed to determine whether the animals became diabetic. iCasp-3.8 mice became diabetic indicating that the administration of the drug induced caspase-mediated death in a significant proportion of the islets in some mice (Fig 1d). The iCasp-3.4 line did not become diabetic upon drug treatment and therefore was chosen to study the immune response to tissue apoptosis *in vivo*. To examine apoptotic cell death, pancreatic sections from iCasp-3.4 mice were analyzed for TUNEL staining in the islets at different time points after drug treatment. TUNEL positive cells were observed 4 hours after drug administration and peaked at 8 hours, indicating that A20187 is able to induce apoptosis in the β-islets of the pancreas shortly after treatment (Fig 1e).

In order to determine whether the antigens from apoptotic cells were cross-presented to T cells, other transgenic models were also used. RIP-gp mice express the lymphocytic choriomeningitis virus (LCMV) glycoprotein (gp) under the control of the rat insulin promoter. This provided a defined islet specific gp antigen that would allow us to ask whether this antigen could be detected by T cells after the induction of apoptosis. Therefore RIP-iCasp-3.4 transgenic mice were bred with RIP-gp transgenic mice in order to create RIP-iCasp3.4/gp mice. P14 transgenic mice express a T cell receptor (TCR) that recognizes the LCMV-gp [24]. For our studies we adoptively transferred CD90.1 congenic (or CD45.1 congenic) CFSE-labeled P14 T cells into RIP-gp or RIP-iCasp3.4/gp and then subsequently injected the mice with AP20187 the following day. Five days post-AP20187 injection we assessed proliferation of the transferred cells in the pancreatic draining lymph node (PDLN) as well as a control non-draining LN, the inguinal lymph node (ILN). Fig 1f shows that increased P14 T cell proliferation was observed in the draining lymph node after the induction of islet apoptosis, suggesting that islet apoptosis via AP20187 injection results in the release of tissue-specific gp-antigen which is presented to T cells in the draining lymph node. The proliferation of P14 cells in RIP-iCasp3.4/gp mice not treated with AP20187 was similar to that seen in RIP-gp mice demonstrating that there was little or no spontaneous caspase-3 activation and release of antigens in the absence of drug-induced dimerization of the transgene.

We next assessed the role of CD11c+ cells in the cross-presentation of antigen from apoptotic islet cells. The RIP-iCasp3.4/gp mice were crossed onto mice expressing the diphtheria toxin receptor under the CD11c promoter (CD11c-DTR). This allowed us to selectively deplete CD11c+ cells in RIP-iCasp3.4/gp mice. P14 proliferation was used to readout antigen-presentation after apoptosis was induced in the absence of CD11c+ cells. The depletion of CD11c+ cells significantly reduced the proliferation of P14 T cells, demonstrating that CD11c+ cells are the main cell population for cross-presenting antigen in this system (Fig 1g).

### P14 T cells produce IFN-γ in response to islet apoptosis but fail to induce diabetes

**Fig 2:**
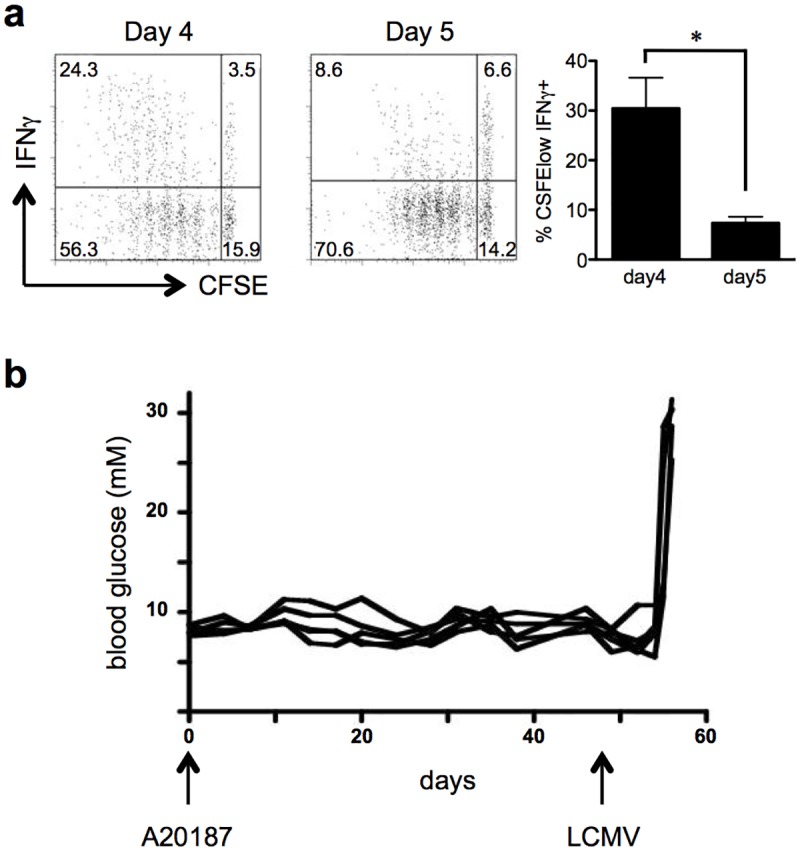
Proliferation and transient IFN-γ production by T cells responding to antigens from apoptotic cells. 3x10^6^ CFSE-labeled P14 T cells were transferred into RIP-iCasp3.4/gp mice on day -1. On day 0 the mice received AP20187. (a) CFSE dilution and IFN-γ production by P14 T cells in the PDLN of mice were assessed on days 4 and 5 after AP20187 injection. (b) Blood glucose was monitored for 49 days after AP20187 injection. On day 49, the mice were injected with LCMV-Armstrong and blood glucose was monitored for an additional 11 days. Experiments were performed 3 times (a) and conducted with 10 mice (b). Error bars in bar graph show S.E.M. (*p < 0.05).

In order to address whether the cross-presentation of sequestered tissue antigen could lead to full T cell activation, we evaluated P14 T cell activation in RIP-iCasp-3.4/gp mice following AP20187 treatment. The induction of apoptosis was sufficient to invoke proliferation of P14 T cells in the PDLN, which was associated with an ability of the P14 cells to produce IFN-γ upon peptide stimulation. Notably, the frequency of IFN-γ production on day 5 was reduced compared to production on day 4 (Fig 2a). These data clearly demonstrate that a small burst of antigen release from the pancreas is sufficient to promote CD8 T cell activation as measured by their ability to proliferate and produce IFN-γ. Surprisingly, although we were able to detect T cell proliferation and IFN-γ production by P14 T cells we never observed the induction of diabetes in these mice even when we tracked blood glucose for over 40 days (Fig 2b). In order to ensure that the mice were still susceptible to gp-dependent tissue destruction we infected them with LCMV on day 49 and monitored blood glucose until day 60. 100% of the mice became diabetic after LCMV infection with similar kinetics to control RIP-gp mice infected with LCMV. This indicates that the reason why mice did not develop diabetes after β-cell apoptosis was not due to altered gp expression on the islets, impaired T cell function nor a dominant regulatory response.

### Recognition of antigen derived from apoptotic cells induces tolerance in responding CD8 T cells

**Fig 3:**
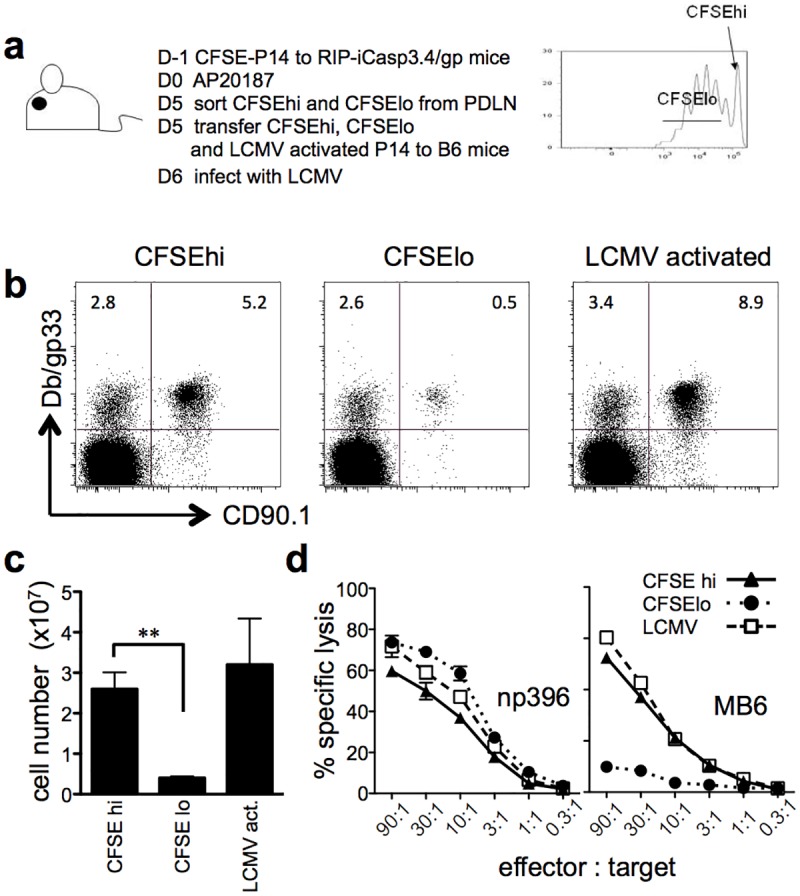
T cells exposed to cross-presented antigen from apoptotic cells are tolerant. (a) Schematic representation of the experiment. CFSE-labeled CD45.1+ P14 T cells were transferred to RIP-iCasp3.4/gp mice followed by AP20187 injection the next day. P14 T cells that were stimulated with apoptotic antigens (CFSE^lo^) or P14 cells that did not encounter antigen (CFSE^hi^) in the PDLN of AP20187 treated RIP-iCasp3.4/gp mice were sorted 5 days after drug injection and transferred to naïve B6 mice. CD8+ T cells from LCMV-infected P14 mice were also transferred into naïve B6 mice. The next day recipient mice were infected with LCMV and analyzed 8 days post-infection. (b) Frequency and (c) number of transferred P14 cells in the spleens of LCMV infected animals. Dot plots are gated on CD8+ cells. (d) NP396 and MB6 specific cytotoxic T cell activity in the spleens of infected mice was evaluated using an *ex vivo* CTL assay. MB6 is a peptide specifically recognized by the P14 TCR but not by endogenous gp33-specific CD8 T cells. Data are representative of 2 independent experiments and error bars show S.E.M. (**p < 0.01).

Since diabetes was not induced in RIP-iCasp3.4/gp mice, we wanted to determine whether T cells were tolerized or fully activated to exposure of apoptotic antigen. Tolerance of CD8 T cells is associated with proliferation prior to deletion of the cells [27] and therefore we examined the fate of the P14 T cells that proliferated in response to cross-presented apoptotic antigen. On average, 50% of the P14 T cells in the draining lymph node remained undivided 5 days following the induction of apoptosis and thus presumably had not been exposed to antigen. We sorted both the antigen-exposed (CFSE^lo^) and naïve (CFSE^hi^) cells from the draining lymph nodes of RIP-iCasp3.4/gp mice and retransferred an equal number of cells to naïve B6 recipients. An additional control group of B6 mice were included which received P14 T cells from mice infected with LCMV. The day after T cell transfer, recipient mice were infected with LCMV and analyzed 8 days later for their ability to mount a secondary response (Fig 3a). LCMV infection led to a robust expansion of the transferred naïve, CFSE^hi^ P14 cells and LCMV activated-P14 cells compared to the CFSE^lo^ P14 cells (Fig 3b and 3c). We then measured the cytolytic activity of the transferred P14 cells using the MB6 peptide which is specifically recognized by the P14 TCR but not by endogenous gp33-specific cytotoxic T lymphocytes (CTLs) [28]. Strikingly, mice that received CFSE^lo^ P14 cells which were previously exposed to apoptotic antigen had about a 30-fold reduction in killing activity. The endogenous response to np396 served as a control to show that there was no detectable difference amongst mice receiving CFSE^hi^ cells, CFSE^lo^ cells or LCMV-activated cells (Fig 3d). These results demonstrate that T cells which encountered antigens derived from apoptotic cells undergo initial rounds of proliferation. Following transfer into naïve mice and subsequent LCMV challenge, however, T cells that have encountered antigens released by apoptosis showed a limited effector function compared with naive CFSE^hi^ T cells, or LCMV-activated effector T cells. These data demonstrate that T cells exposed to antigens derived from apoptotic cells are not able to obtain strong effector function upon LCMV infection.

### Additional stimuli that enhance innate and adaptive immunity do not result in diabetes

**Fig 4:**
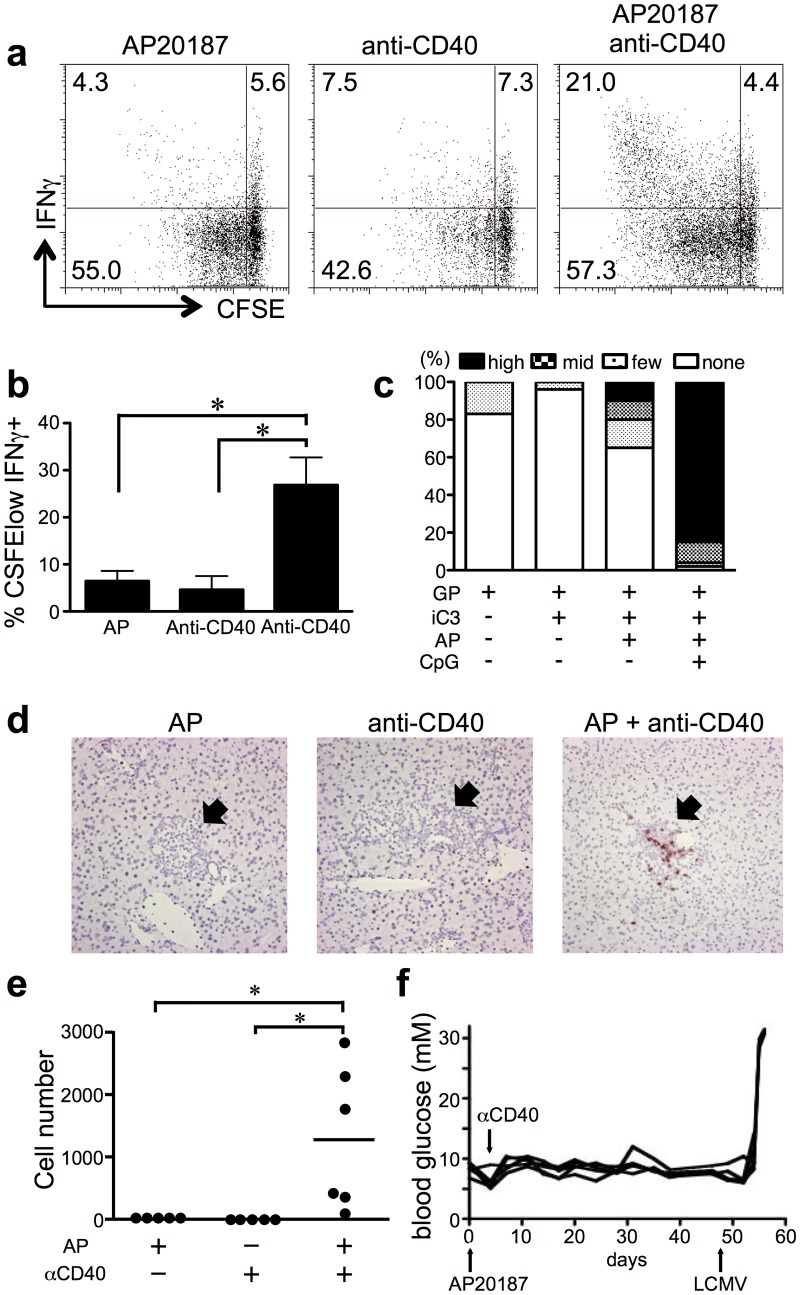
APC maturation signals promote T cell activation and inflammation, but are unable to induce diabetes. (a) and (b) CFSE-labeled P14/CD45.1+ T cells were transferred to RIP-iCasp-3.4/gp mice and treated with AP20187. Anti-CD40 antibody was injected 2 days later. After 3 days, cells from the PDLN were analyzed for division by CFSE dilution and IFN−γ production. (c) and (d) CD8 T cell infiltration in the pancreas was evaluated by histology sections which were stained with anti-CD8. (e) Infiltrating P14 T cells and CD8+ T cells in the pancreas were quantified. (f) P14 T cells were transferred into RIP-iCasp-3.4/gp mice. After 24 hours, AP20187 was given i.p., followed by anti-CD40 antibody i.v. 2 days later. Blood glucose levels were monitored every other day for 60 days. At day 49, mice were infected with LCMV-Armstrong. Data are representative of at least 5 mice per condition from independent experiments and error bars in bar graph show S.E.M. (*p < 0.05).

Current models predict that the signals to induce APC maturation are critical for promoting the full activation of T cells. Therefore, apoptosis was induced in the presence of anti-CD40 and/or CpG DNA to determine whether this would alter the fate of T cells and lead to enhanced T cell function and autoimmunity. P14 T cells were adoptively transferred into RIP-iCasp3.4/gp mice, which were then treated with AP20187 and an agonist monoclonal antibody against CD40. Interestingly, enhanced proliferation was observed on day 5 when anti-CD40 antibody was added two days after AP20187 injection. Importantly, anti-CD40 combined with apoptosis resulted in strong IFN-γ production in P14 T cells (Fig 4a and 4b). Together these results suggest that inflammatory stimuli such as anti-CD40 can enhance the effector function of T cells upon the release of antigens from apoptotic cells.

Upon observing the enhanced activation of P14 T cells in response to antigen in the presence of anti-CD40 we questioned whether the cells were now capable of infiltrating the pancreas and causing diabetes. Immunohistochemical analysis of pancreata from RIP-iCasp-3.4/gp mice showed that P14 T cell infiltration was strongly increased in mice treated with AP20187 together with CpG or anti-CD40 (Fig 4c and 4d). We also calculated the number of P14 cells in the pancreas by digesting the tissue and performing flow cytometry. The results indicated that only antigen exposure accompanied by inflammatory signals induced infiltration of P14 cells into the pancreas (Fig 4e). Importantly, although antigen exposure combined with anti-CD40 or CpG could promote CD8 infiltration into the islets and prolonged IFN-γ production, diabetes was not induced (Fig 4f). As a control, these mice were infected with LCMV which resulted in the induction of autoimmunity in these animals. Together, these data demonstrated that encountering apoptotic antigens in the context of additional signals that promote T cell function is still insufficient to result in autoimmunity, despite the infiltration of T cells into the organ.

**Fig 5:**
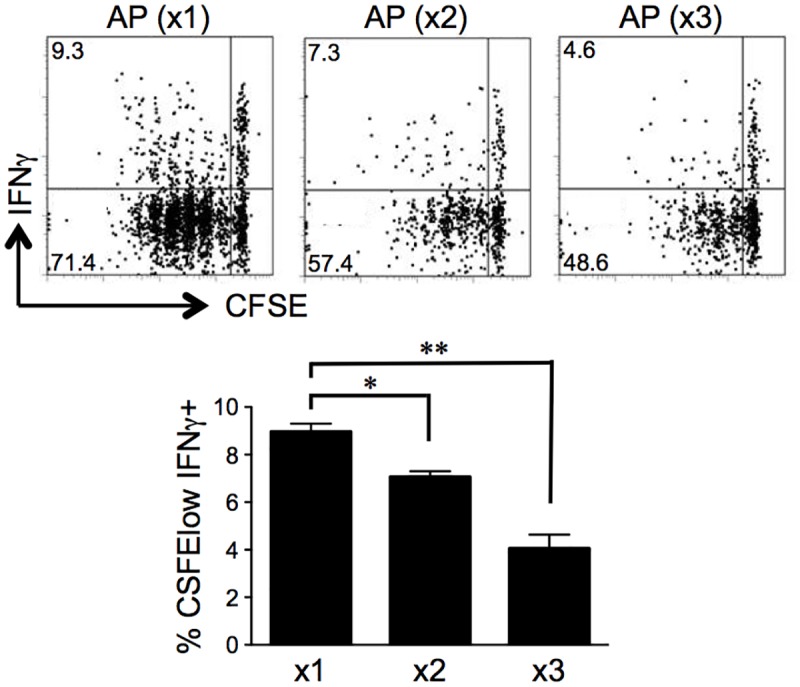
Repeated exposure to antigen from apoptotic cells induces T cell death. RIP-iCasp3.4/gp mice which received CFSE-labeled P14 T cells on day -1 were injected with AP20187 3 times (days -4, -2, 0), 2 times (days -2, 0) or once (day 0). Proliferation and IFN-γ production by the transferred cells was assessed in the draining lymph node on day 5. Data are representative of at least 3 mice per condition from independent experiments. Error bars in bar graph show S.E.M. (*p < 0.05, **p < 0.01).

In order to increase the duration of antigen presentation, we induced apoptosis by infusing AP20187 either three times, twice or once in RIP-iCasp3.4/gp mice. Fig 5 shows that inducing apoptosis multiple times reduces the number of proliferating P14 cells in the draining lymph node on day 5, suggesting that increasing the duration of antigen exposure was not sufficient to improve T cell activation. In fact, it appeared that repeated antigen exposure in the absence of additional DC maturation signals accelerated the deletion of autoreactive CD8 T cells.

**Fig 6:**
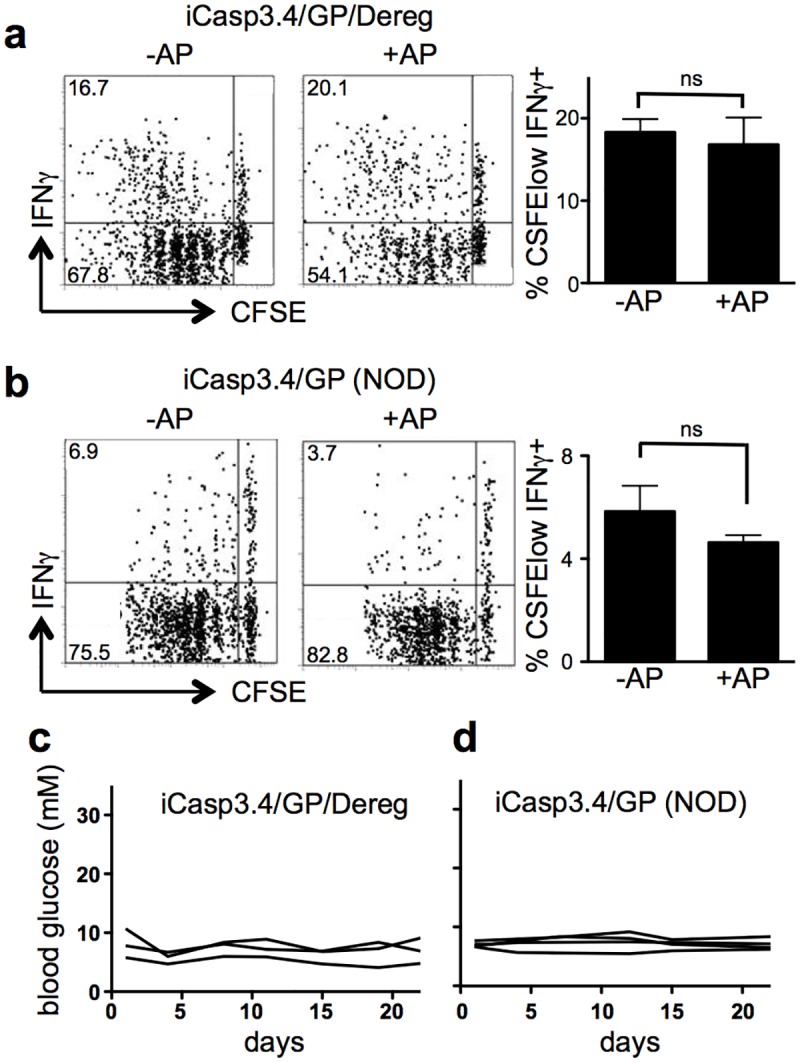
Tregs and the host genetic background do not alter the response of autoreactive T cells. (a) RIP-iCasp3.4/gp/DEREG mice were depleted of Tregs on days -2, 0, and +2. CFSE-labeled P14 T cells were transferred on day -1 and AP20187 administered on day 0. Proliferation and IFN-γ production were assessed in the draining lymph node on day 5. (b) CFSE-labeled P14 NOD T cells were transferred into RIP-iCasp3.4/gp mice on the NOD background followed by AP20187 administration one day later. Proliferation and IFN-γ production in the transferred cells were assessed 5 days after drug infusion. (c) and (d) Blood glucose was monitored after AP20187 injection. Data are representative of at least 3 mice per condition from independent experiments and error bars in bar graphs show S.E.M.

Regulatory T cells (Tregs) are a critical cell population for controlling immunological tolerance by suppressing APC functions and T cell activation [29, 30]. Thus we questioned whether depletion of Tregs combined with the induction of apoptosis and subsequent antigen-presentation would be sufficient to lead to autoimmune diabetes. To this end, we crossed the RIP-iCasp3.4/gp mice to DEREG (Depletion of REGulatory T cells) mice, which express the diphtheria toxin receptor under the Treg-specific Foxp3 promoter. For these studies, we depleted Tregs in RIP-iCasp3.4/gp/DEREG mice by injection of diphtheria toxin 1 day prior to the adoptive transfer of CFSE-labeled P14 T cells. We then injected AP20187 in order to induce islet apoptosis and then assessed the proliferation and cytokine production of the transferred cells in the draining lymph node 5 days later. To our surprise we found that even in the absence of apoptosis we could clearly detect the proliferation of the P14 T cells in the draining lymph node of Treg-depleted mice (Fig 6a). This is in contrast to RIP-gp mice where there is much less proliferation under the same conditions (Fig 1f). The induction of β-islet apoptosis did not enhance either the proliferation or cytokine production of P14 cells in the absence of Tregs (Fig 6a) suggesting that the loss of Tregs is sufficient to allow the cross-presentation of low levels of tissue antigen. However, the deletion of Tregs was unable to promote a sufficient autoimmune response that resulted in autoimmune diabetes (Fig 6c).

The C57Bl/6 strain of mice is relatively resistant to the development of autoimmunity. In contrast, the NOD strain is autoimmune-prone and mice naturally develop diabetes over time. One of the factors thought to play an important role in the initiation of diabetes in the NOD strain is a wave of apoptosis that occurs in neonatal mice [16–18]. We wanted to determine whether the genetic background of the mice could influence whether antigen release was sufficient to lead to diabetes in this model. To accomplish this we crossed the P14 TCR onto the NOD strain (12 backcrosses) and also crossed the RIP-iCasp3.4 and RIP-gp onto the same background. Unlike what we observed on the B6 background, but similar to Treg-depleted mice, P14 T cells spontaneously proliferated in the draining lymph node of NOD RIP-gp mice even in the absence of apoptosis (Fig 6b). The induction of apoptosis had little impact on this proliferation or cytokine production. In addition, NOD RIP-iCasp3.4/gp mice did not display any hyperglycemia even when we tracked the mice for nearly 4 weeks after the induction of apoptosis (Fig 6d). Therefore, the amount of antigen available in RIP-gp mice on the NOD background is not limiting and increasing the amount of antigen by inducing apoptosis in the presence of autoreactive T cells does not induce the development of diabetes.

## Discussion

In the hit-and-run model of autoimmunity, an initial event such as tissue damage is proposed to be sufficient to cause the release of self-antigen and the subsequent development of a self-sustaining autoimmune T cell response. Here we described a model which has allowed us to address the fundamental question of whether the controlled transient exposure of CD8 T cells to self-antigen *in vivo* can lead to autoimmune diabetes. The advantage of our model is that we are able to control self-antigen presentation both spatially (within the lymph node draining the target organ) and temporally *in vivo*. By generating a transgenic mouse model that allowed us to induce apoptosis in a single defined tissue expressing a model self-antigen, we were able to demonstrate that the release of self-antigen can be cross-presented in the lymph nodes by CD11c+ cells without inducing autoimmune diabetes. Recently it was reported that the intra-islet APCs are F4/80+ CD11c+ CD11b+ MHCII+ macrophages and a few CD103+ DCs, which are essential for autoimmune diabetes development [31]. Thus, the CD11c+ cells we observed could be macrophages or DCs and it would be important to determine which population of CD11c+ cells are detected within the islets and the lymph nodes before and after the induction of apoptosis. Melli et al. demonstrated in their study that inflammation mediated by islet-specific CD4 or CD8 T cells was associated with an increase in mature APCs that could enter the draining lymph node [32]. In contrast to our model, the inflammation induced cross-presentation of islet antigens to T cells in their study was associated with T cell activation rather than tolerance. The difference between our results is likely due to the fact that Melli used fully active autoreactive CD4 T cells for the induction of inflammation in the pancreas. Since it is known that CD4 help can prevent the normal process of cross-tolerization of islet-specific T cells by APCs [33] these results are not surprising.

Our model system allowed us to test the impact of CD8 T cells exposed to self-antigen in a sterile environment in the absence of inflammation. We demonstrated that the CD8 T cells underwent a transient activation that was associated with proliferation and production of IFN-γ but these cells were eventually deleted upon repeated antigen exposure. Our results were somewhat surprising, since previous work in another model has demonstrated that CD8 T cells undergoing deletional tolerance do not acquire effector functions such as IFN-γ production [34]. However, our results are consistent with others that have demonstrated that CD8 T cells undergoing deletion express IFN-γ [35].

When we addressed whether the genetic background of the mice controlled aspects of the immune response to apoptotic cells we found that even in the autoimmune-prone NOD background antigen exposure did not lead to autoimmune diabetes. P14 cells transferred into RIP-gp mice on the NOD background unexpectedly underwent spontaneous proliferation in the draining lymph nodes of the mice even without apoptosis induction. This may be due to inflammation already present in the β-islets resulting in more β-islet death compared to B6 mice or perhaps due to the fact that NOD mice have been reported to have a defect in their ability to clear apoptotic cells which should lead to secondary necrosis and subsequent release of inflammatory mediators [36]. In our hands, the induction of apoptosis was not sufficient to promote autoimmune diabetes in the NOD background suggesting that the development of autoimmune diabetes is not limited by a lack of antigen presentation in the draining lymph nodes in this model.

In the absence of Tregs, autoimmune diabetes also did not occur upon apoptosis induction in iCasp3.4/gp/DEREG mice. We noted that the depletion of Tregs led to spontaneous proliferation of P14 T cells in the draining lymph node even in the absence of apoptosis. Since apoptosis occurs spontaneously in most tissues, Tregs might suppress the response to spontaneous cross-presented autoantigens in the steady state. NOD mice have been shown to have many defects including alterations in their Treg cells [37]. Interestingly, RIP-gp mice on the NOD background and C57Bl/6 RIP-gp mice that were depleted of Tregs appeared similar in their constitutive ability to present antigen to P14 T cells in the draining lymph nodes. Previous work has shown that Tregs can control DC homeostasis, with the depletion of Tregs resulting in an increase in the number of DCs in the lymphoid organs [38]. In addition to an increase in DC numbers upon Treg depletion, another study has demonstrated that Treg depletion also increases the expression of co-stimulatory molecules on the DCs which increases their stimulatory capacity [39]. Several studies showed that engulfment of apoptotic cells induces phagocytes to produce an immunosuppressive cytokine, TGF-β, which plays an important role in peripheral differentiation of Treg cells [14, 40–42]. Further study of Treg differentiation and movement in our model might elucidate the tissue homeostasis of Treg.

Overall, our studies suggest that the recognition of antigen from apoptotic cells generally leads to proliferation and the induction of tolerance in autoreactive CD8 T cells. Current models suggest that the release of self-antigen from apoptotic cells could be immunogenic in the presence of inflammatory signals such as DAMPs or pathogen-associated molecular patterns (PAMPs). In this study we used anti-CD40 and CpG (a TLR9 ligand) as inflammatory stimuli and both inflammatory conditions did not result in autoimmune diabetes upon apoptosis induction. It is still possible that other stimuli such as DAMPs might have an ability to augment tissue destruction. However, there is evidence which shows that certain receptors for apoptotic cells play a role in maintaining the tolerant state of the DCs by altering intracellular signalling pathways [43]. Also phagocytes which engulf apoptotic cells secrete TGF-β, IL-10 and prostaglandin E2 to suppress inflammation [14, 40, 44]. Thus, tolerance is positively regulated by certain signals derived from apoptotic cells, and that may not be easily overcome by the induction of inflammatory conditions or in the autoimmune-prone background NOD mice. The ability of other forms of cell death, including necroptosis and pyroptosis, to induce autoimmunity *in vivo* has not yet been addressed. However, the release of DAMPs from necroptosis has been considered to trigger an inflammatory response and has been associated with inflammatory diseases [45, 46]. Thus, other type of cell death might be capable of initiating an autoimmune response.

One question we are currently addressing is whether antigen from apoptotic cells can activate autoreactive CD4 T cells and whether this is sufficient to drive autoimmunity. In the absence of CD4 help, apoptotic cells have been shown to induce TRAIL-mediated tolerance in CD8 T cells [11]. Future studies will be important to determine whether the presence of autoreactive CD4 T cells is sufficient to promote the activation of CD8 T cells in response to small amounts of tissue antigen. Our preliminary studies suggest that the presentation of antigens from apoptotic cells to CD4 T cells does not occur very effectively *in vivo*. However, it will be interesting to determine how autoreactive CD4 T cells are prevented from being activated by the presentation of antigens derived from apoptotic cells as this likely prevents the subsequent activation of autoreactive CD8 T cells and autoimmunity.
